# Two Ellagic Acids Isolated from Roots of *Sanguisorba officinalis* L. Promote Hematopoietic Progenitor Cell Proliferation and Megakaryocyte Differentiation

**DOI:** 10.3390/molecules19045448

**Published:** 2014-04-24

**Authors:** Xiaoping Gao, Jianming Wu, Wenjun Zou, Yanping Dai

**Affiliations:** 1 College of Pharmacy, Chengdu University of Traditional Chinese Medicine, Chengdu, 86611-137, China; E-Mails: wjmbj@126.com (J.W.); aiyanping198909@163.com (Y.D.); 2 School of pharmacy, Guiyang Medical College, Guiyang, 86550-004, China

**Keywords:** *Sanguisorba officinalis* L, 3,3',4-tri-*O*-methylellagic acid 4'-*O-β*-d-xyloside, 3,3',4-tri-*O*-methylellagic acid, hematopoietic progenitor cells, cell proliferation, megakaryocyte differentiation

## Abstract

Using a bioassay-directed chromatographic separation, two ellagic acids were obtained from the ethyl acetate extract of the roots of *Sanguisorba officinalis* L. On the basis of chemical and spectroscopic methods, the two ellagic acids were identified as 3,3',4-tri-*O*-methylellagic acid-4'-*O-β*-d-xyloside and 3,3',4-tri-*O*-methylellagic acid. Stimulation of cell proliferation was assayed in hematopoietic progenitor cells using the Cell Counting kit-8 method. The megakaryocyte differentiation was determined in human erythroleukemia (HEL) cells using Giemsa staining and flow cytometry analysis. The ellagic acids significantly stimulated the proliferation of Baf3/Mpl cells. Morphology analysis and megakaryocyte specific-marker CD41 staining confirmed that the ellagic acids induced megakaryocyte differentiation in HEL cells. This is the first time that 3,3',4-tri-*O*-methylellagic acid or 3,3',4-tri-*O*-methylellagic acid-4'-*O-β*-d-xyloside are reported to induce megakaryopoiesis, suggesting a class of small molecules which differ from others non-peptidyl, and appears to have potential for clinical development as a therapeutic agent for patients with blood platelet disorders.

## 1. Introduction

The induction of megakaryocyte differentiation is a potent strategy for the clinical treatment of diseases related to blood platelet disorders. Thrombopoietin (TPO) and its receptor (c-Mpl) are important regulators of platelet production. TPO binds to the c-Mpl on megakaryocytic progenitors and stimulates proliferation and differentiation leading to increased platelet production. Recombinant human TPO have been shown to be potent stimulator of megakaryocyte growth and platelet production in several clinical trials [1,2], but an immune response and the generation of anti-TPO antibodies impede the development of recombinant TPO [3,4]. Current and future interest is focused on the development of TPO mimetics, especially non-peptidyl molecules [5,6].

*Sanguisorba officinalis* L. is a perennial plant widely distributed in China, and its roots have been used as a Traditional Chinese Medicine for the treatment of myelosuppression caused by chemotherapy or radiotherapy, especially in patients with leucopenia [7,8,9,10]. We have recently reported that the ethyl acetate extract of *Sanguisorba officinalis* L. promotes proliferation of Baf3/Mpl cells in the absence of TPO, and induces megakaryocyte differentiation [11,12]. The extract contains a variety of chemical constituents, including triterpenoids (approximately 70% of the extract) and phenolics (approximately 30% of the extract), but the major chemical constituents exerting the effect of megakaryopoiesis remain to be identified. We therefore attempted to isolate and identify the major constituents by bioactivity-guided methods. Two compounds **1** and **2** were thus obtained, and their stimulatory effects of cellular proliferation and megakaryopoiesis were evaluated in Baf3/Mpl cells and human erythroleukemia (HEL) cells, respectively.

## 2. Results and Discussion

### 2.1. Structural Determination of the Compounds **1** and **2**

**Figure 1:**
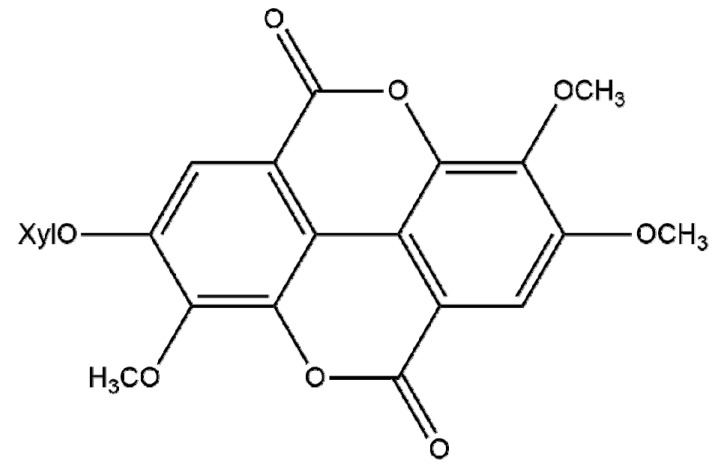
Structure of compound **1**.

Compound **1** was obtained as a colorless amorphous powder. The ESI-MS spectrum displayed a molecular ion peak [M+H]^+^ at *m/z* 477, and peaks at 344 [M−132]^−^ and 313 [344−31]^−^ suggested the presence of a pentose and methoxyl, consistent with a molecular formula of C_22_H_20_O_12_ with 13 degrees of unsaturation. The ^1^H-NMR spectrum (DMSO-d_6_) showed the presence of two aromatic protons at *δ* 7.74 (s, 1H) and *δ* 7.51 (s, 1H), three methoxyl signals at *δ* 4.08 (s, 3H), *δ* 4.03 (s, 3H) and *δ* 3.98 (s, 3H), and one glycosyl signals at *δ* 5.16 (d, 1H, *J* = 7 Hz). The ^13^C-NMR spectrum (DMSO-d_6_) showed two benzene rings and two cyclic lactone signals at *δ* 158.42, *δ* 158.31 (Table 1), characteristic of an ellagic acid. The ^1^H- and ^13^C-NMR spectra of compound **1** were identical with those reported in literature data [13,14], therefore, the structure of compound **1** was determined as 3,3',4-tri-*O*-methylellagic acid 4'-*O-β*-d-xyloside (Figure 1).

**Table 1:** NMR Spectra of Compounds **1**–**2** [600 and 160 MHz, DMSO- d_6_, δ (ppm), *J* in Hz].

Atom	Compound 1		Compound 2	
	^1^H	^13^C	^1^H	^13^C
1		111.13		111.62
2		141.40		141.23
3		140.71		140.26
4		152.57		152.23
5	7.74	111.66	7.73	111.42
6		111.83		112.11
7		158.42		158.52
1′		112.43		111.65
2′		141.42		141.25
3′		140.15		140.23
4′		153.73		152.22
5′	7.51	107.44	7.61	111.45
6′		113.20		112.12
7′		158.31		158.63
3-OMe	4.08	60.63	4.19	61.53
3'-OMe	4.03	61.22	4.14	61.26
4-OMe	3.98	56.65	4.04	56.51
4'--Xyl	5.16 (*J* = 7)			

**Figure 2:**
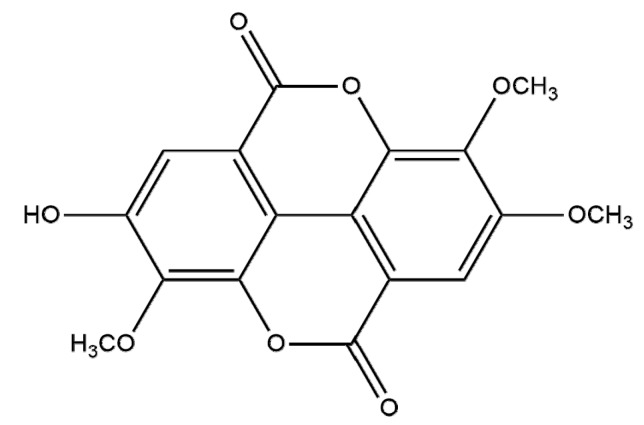
Structure of compound **2**.

Compound **2** was obtained as a pale yellow amorphous powder which produced a positive reaction to FeCl_3_ reagent. The ESI-MS spectrum of compound **1** displayed a molecular ion peak at *m/z* 345 [M+H]^+^ and a peak at *m/z* 343 [M−H]^−^, consistent with a molecular formula of C_17_H_12_O_8_, with 12 degrees of unsaturation. The ^1^H-NMR spectrum (DMSO-d_6_) showed the presence of two aromatic protons at *δ* 7.73 (s, 1H) and *δ* 7.61 (s, 1H), three methoxyl signals at *δ* 4.19 (s, 3H), *δ* 4.14 (s, 3H) and *δ* 4.04 (s, 3H). The ^13^C-NMR spectrum (DMSO-d_6_) showed two asymmetric benzene rings, three methoxyl signals at *δ* 61.53, *δ* 61.26 and *δ* 56.51 (Table 1). Comparison with of the ^1^H and ^13^C-NMR spectra with those of compound **1**, indicated they were similar except for the absence of the glycosyl signal in **1** [15]. Therefore, the structure of compound **2** was elucidated as 3,3',4-tri-*O*-methylellagic acid (Figure 2). 3,3',4-tri-*O*-Methylellagic acid-4'-*O-β*-d-xyloside and 3,3′,4-tri-*O*-methylellagic acid were this both determined to be known compounds, the latter having been identified in 13 plants (Table 2).

**Table 2:** The plants containing 3,3',4-tri-*O*-methylellagic acid.

Series	The plants containing 3,3',4-tri-*O*-methylellagic acid
1	*Euphorbia hylonoma* Hand-Mazz [16]
2	*Irvingia gabonensis* [17]
3	*Phyllagathis rotundifolia* [18]
4	*Potentilla candicans* [19]
5	*Euphorbia antiquorum* L. [20]
6	*Eucalyptusglobulus* Labill [21]
7	*Polygonum runcinatum* Buch [22]
8	*Sapum sebiferun* (L.) Roxb. [23]
9	*Euphorbia pekinensis* Rupr [24]
10	*Phyllanthus emblica* L. [25]
11	*Euphorbia soongarica* Boiss [26]
12	*Dipentodon sinicus* [27]
13	*Sonneratia caseolaris* [28]

### 2.2. Compounds **1** and **2** Stimulated Cell Proliferation

**Figure 3:**
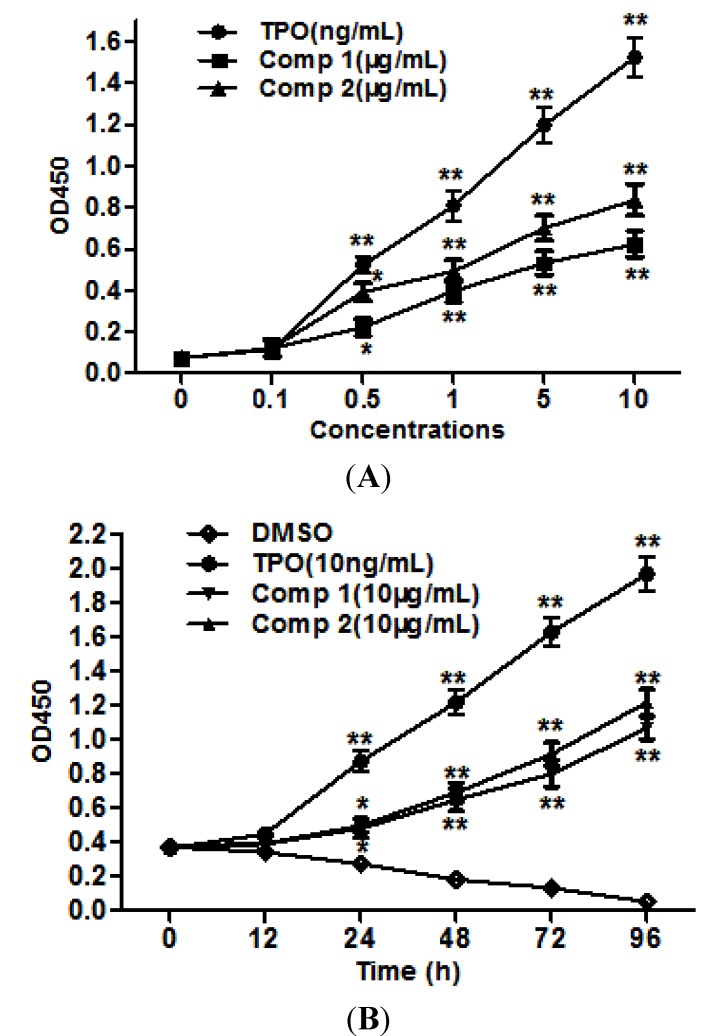
Compounds promote the proliferation of Baf3/Mpl cells. (**A**) Compounds **1** and **2** stimulate cell proliferation in a dose-dependent manner. (**B**) Compounds **1** and **2** stimulate cell proliferation in a time-dependent manner. Results are representative of at least three independent experiments run in triplicate and expressed as the mean ± SD.

To confirm the megakaryopoietic effects, we measured first cell-proliferative activity of two compounds in Baf3/Mpl cells. As shown in Figure 3A,B, compounds **1** and **2** promoted the proliferation of Baf3/Mpl cells in a dose-dependent and time-dependent manner under cytokine-free conditions. These results suggested that compounds **1** and **2** stimulate cell proliferation via TPO-independent or cytokines-independent pathways.

### 2.3. Compounds **1** and **2** Increased Megakaryocyte Ploiy in HEL Cells

**Figure 4:**
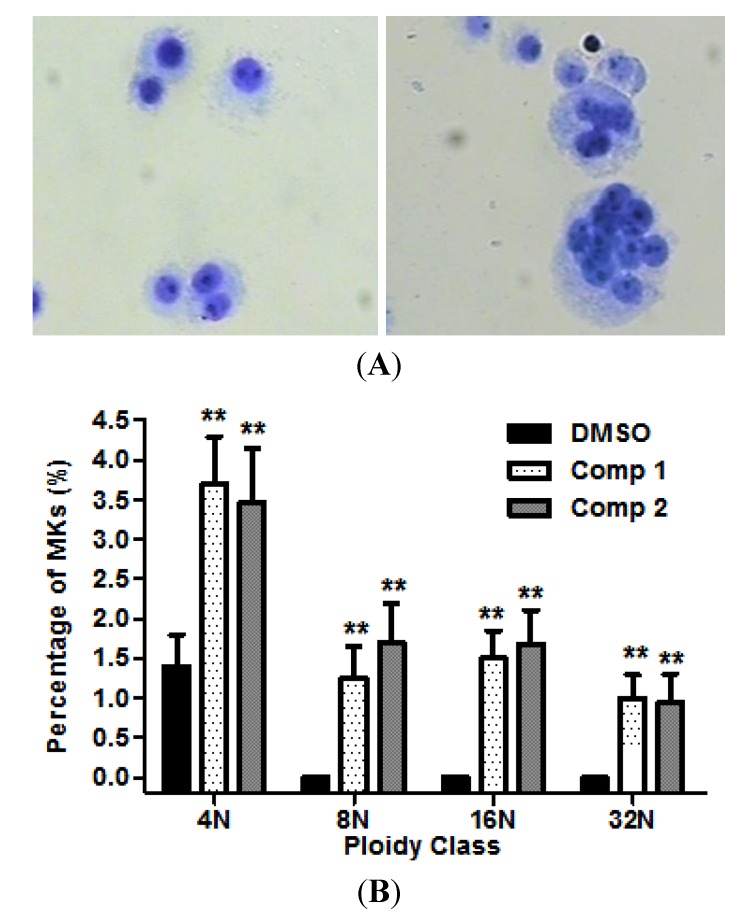
Compounds induced megakaryopoiesis in HEL cells. (**A**) Light microscopy shows that the image of megakarycytes (control and compound **1**, left and right respectively) stained by Giemsa; (**B**) The percentage of megakaryocytes was determined on the basis of the number of endomitosis. Results are representative of at least three independent experiments run in triplicate and expressed as the mean ± SD.

We next examined whether the compounds possess the capacity to induce megakaryocyte differentiation. HEL cells were cultured for 12 days in serum-free medium containing 20 μg/mL compounds **1** or **2**, DMSO as a negative control. As shown in Figure 4A, Giemsa staining clearly showed a morphological change of polyploidy megakaryocytes (right panel). The polyploidy megakaryocyts were determined by counting the number of endomitotic cells. The mean ploidy was increased from 1.35% to 7.35%, having a tendency to increase the megakaryocytes from 8N to 32N compared with control (Figure 4B).

### 2.4. Flow Cytometric Analysis

**Figure 5:**
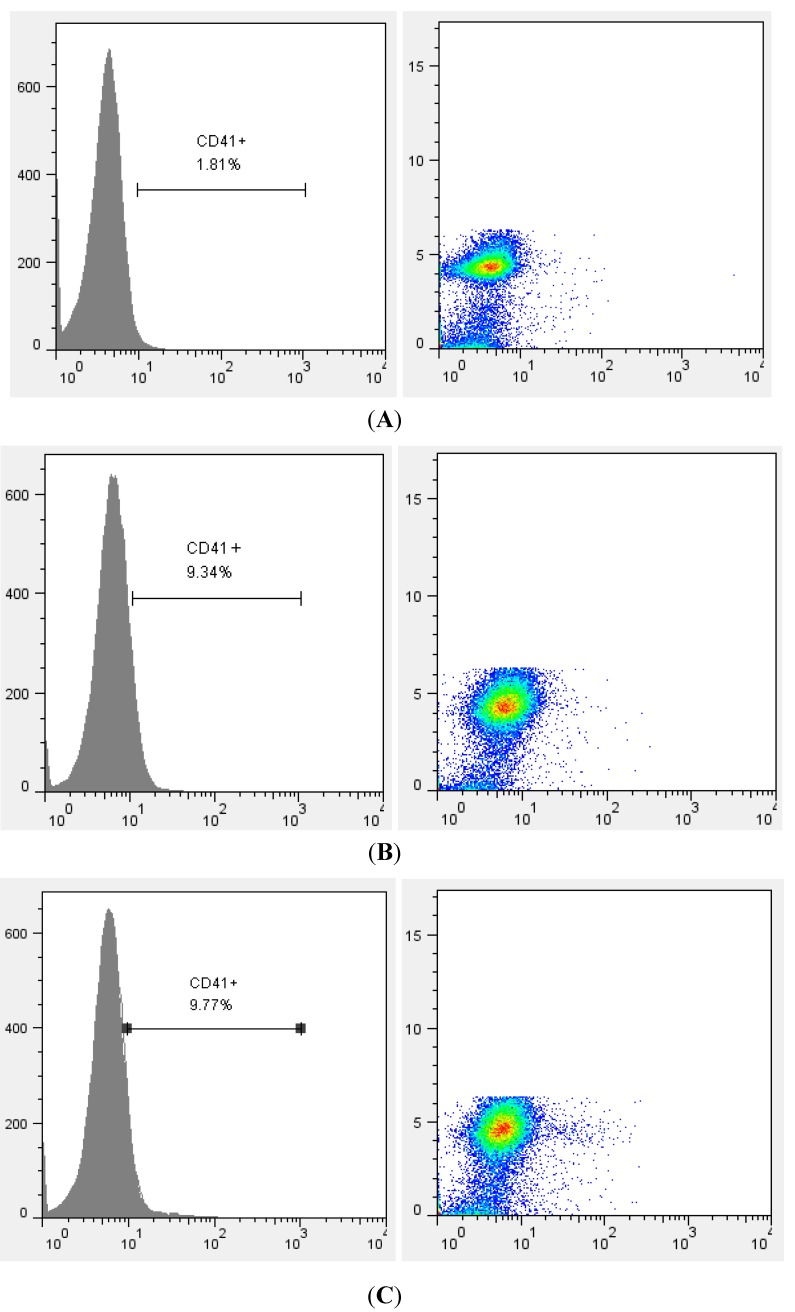
Flow cytometric analysis of HEL cells stained with anti-CD41-PE. Histogram plot (left panel) and dot plot (right panel) show the percentage of CD41^+^ cells. Data represent one experiment of two independent experiments. (**A**) Control (DMSO); (**B**) Compound **1** (20 μg/mL); (**C**) Compound **2** (20 μg/mL).

The expression of CD41 (GPIIb/IIIa), which is considered to be an early megakaryocyte-specific marker (MK-specific marker), was analyzed by flow cytometry. Corresponding to the result of Giemsa staining, both compounds 1 and 2 increased the expression of MK-specific marker CD41 (Figure 5). These results indicate that the compounds have the potential to induce megakaryocyte differentiation and maturation *in vitro*.

## 3. Experimental

### 3.1. General Information

The NMR spectra (^1^H, ^13^C) were recorded on a Bruker AV- 600 spectrometer (Bruker Biospin AG, Fällanden, Switzerland, 600.13 for ^1^H and 159.91 MHz for ^13^C), with DMSO-d_6_ as solvent and tetramethylsilane (TMS) as reference. Mass-spectrometric (MS) analysis was performed using a Bruker BioTOF Q mass spectrometer (Bruker Daltonics, Billerica, MA, USA). HPLC separation was performed on AKTA Basic (GE Healthcare, Pittsburgh, PA, USA) equipped with a multi-wavelength tunable UV detector , a Frac920 fraction collector (GE Healthcare, Pittsburgh, PA, USA) and Kromasil C_18_ preparative column (250 mm × 20 mm,5 μm). For the cell proliferation assay and flow cytometric analysis, a SpectraMax M5 Microplate Reader (Molecular Devices, Sunnyvale, CA, USA) and Guava easyCyte™ flow cytometer (Merck Millipore, Billerica, MA, USA) were used.

### 3.2. Plant Material

The roots of *Sanguisorba officinalis* L. were purchased from a local market in Chengdu (China), and were identified at Chengdu University of TCM. The voucher specimen was deposited in the laboratory of Chengdu University of TCM.

### 3.3. Extraction and Isolation

The air-dried roots (500 g) were ground, and then reflux-extracted with 75% ethanol three times (3 × 5,000 mL, 1 h each). After filtration and evaporation, the ethanol extract was suspended in water and incubated at 60 °C for 30 min. The ethanol extract was then extracted using 500 mL ethyl acetate three times continuously. The solvent was evaporated under vacuum to yield the ethyl acetate extract of *Sanguisorba* *offoconalis* L. (11.2 g). The ethyl acetate extract (5.0 g) was dissolved in methanol and subjected to AKTA column chromatography, which were separated using Kromasil C_18_ column (250 mm × 20 mm, 5 μm) and eluted at 10 mL/min flow rate and wavelength 254 nm using pH 3.0 hydrochloric acid (solvent A) and MeOH (solvent B) with the following gradient of composition: starting with 20% solvent B and changing to 60% during 35 min, followed by a second ramp to 100% B in 10 min, maintained for 10 min, and then was purified by preparative HPLC to yield compound 1 (8.4 mg, purity 98.5%, RT30.71 min) and compound 2 (14.4 mg, purity 98.1%, RT33.77 min). The yields and purity of two compounds are shown in Table 3.

**Table 3:** The yields and purity of compounds **1**–**2**.

Compound	Yield (‰)	Purity (%)
1	1.7	98.5
2	2.9	98.1

### 3.4. Cell Proliferation Assay

Baf3/Mpl cells were maintained in RPMI 1640 medium supplemented 10% fetal bovine serum and 5 ng/mL human TPO. After 3 days, the cells were washed twice with serum-free medium and were aliquoted into individual well of a 96-well plate. Compound **1** or compound **2** was dissolved in DMSO and diluted in 1640 medium, and added to cells at different concentrations. DMSO (below 1%) and recombinant human TPO (rhTPO) were used respectively as negative and positive controls. All cells were grown in a humidified incubator at 37 °C and 5% CO_2_. After 72 or 96 h, proliferation assay was performed using the Dojindo Cell Counting kit-8 according to the instruction supplied by the manufacturer. Absorbance values (450 nm) were recorded in triplicate using M5 Microplate Reader.

### 3.5. Megakaryocyte Ploidy and Morphological Analysis

HEL cells were incubated in RPMI 1640 medium supplemented 10% fetal bovine serum in the 24-well plate. Compound **1** or compound **2** at final concentrations of 10 μg/mL or 20 μg/mL was added. DMSO (below 1%) was used in the control wells. All cells were grown in a humidified incubator at 37 °C and 5% CO_2_ for 14 days. Cells were collected on the 4th day, 8th day and 12th day, and stained with Giemsa to assess megakayocyte ploid and morphological analysis.

### 3.6. Flow Cytometric Analysis

HEL cells were incubated in RPMI 1640 medium supplemented 10% fetal bovine serum in the 24-well plate. Compound **1** or compound **2** at final concentration of 20 μg/mL was added. DMSO (below 1%) was used in the control wells. After 12 day, cells were collected and resuspended in 300 μL of phosphate-buffered saline (PBS). Cells were stained with a CD41-PE-conjugated monoclonal antibody (Life Technologies, Carlsbad, CA, USA) and were analyzed on a flow cytometer.

### 3.7. Statistical Analysis

The results are expressed as the mean ± SD. Statistical differences were determined using a One-way ANOVA with *p* < 0.05 considered as statistically significant. Statistical analyses were carried out using the software SPSS 17.0 for Windows.

## 4. Conclusions

Two compounds were isolated from the roots of *Sanguisorba* *offoconalis* L., and identified as the known 3,3',4-tri-*O*-methylellagic acid-4'-*O-β*-d-xyloside and 3,3',4-tri-*O*-methylellagic acid. In the present study, we report for the first time that these compounds not only promote proliferation of megakaryocyte progenitor cells, but also induce megakaryocyte differentiation, resulting in polyploidy formation, MK-specific marker CD41 expression of HEL cells. These results suggest the megakaryopoietic effect of ellagic acids isolated from the roots of *Sanguisorba* *offoconalis* L. The induction of megakaryocyte differentiation is a potent strategy for the clinical treatment of diseases related to blood platelet disorders. Based on the structure analysis, the compounds are a class of small molecules which differ from other non-peptidyl molecules [5,6]. Therefore, it will be necessary to investigate the thrombopoiesis effect of the compounds. Research into the mechanism of megakaryopoiesis may also help guide the clinic application of the compounds and *Sanguisorba* *offoconalis* L. as a Traditional Chinese Medicine.
